# Evaluation of lung perfusion by using lung perfusion SPECT and lung CT with breathing synchronization software

**DOI:** 10.1186/s41824-022-00154-1

**Published:** 2022-11-25

**Authors:** Hidenobu Hashimoto, Tsutomu Soma, Sunao Mizumura, Tadashi Kokubo, Rine Nakanishi, Takanori Ikeda

**Affiliations:** 1 Department of Cardiovascular Medicine, Department of Internal Medicine, Faculty of Medicine, Toho University, 6-11-1, Omorinishi, Ota-Ward, Tokyo, 143-8541 Japan; 2 Software Development Department, PDRadiopharma Inc., Tokyo, Japan; 3 Department of Radiology, Faculty of Medicine, Toho University, Tokyo, Japan; 4 Central Department of Radiology, Toho University Omori Medical Center, Tokyo, Japan

**Keywords:** Lung perfusion SPECT, Lung CT, Pulmonary embolism, Software

## Abstract

**Background:**

Lung perfusion using ^99m^Tc-macroaggregated albumin single-photon emission computed tomography (SPECT) and lung computed tomography (CT) is a useful modality for identifying patients with pulmonary artery embolism. However, conformity between SPECT and CT at the bottom of the lung is generally low. This study aims to investigate the progression of conformity between lung perfusion SPECT and lung CT using a breathing synchronization software.

**Methods:**

Among 95 consecutive patients who underwent lung perfusion SPECT and lung CT within 14 days because of suspected pulmonary embolism between June 2019 and August 2020 in department of cardiovascular medicine, we identified 28 patients (73 ± 10 years) who had normal pulmonary artery on contrast lung CT. We compared lung volumes calculated using lung perfusion SPECT and lung CT as gold standard. Visual conformity between lung SPECT and lung CT was scored 0–4 (0: 0–25%, 1: 25–50%, 2: 50–75%, 3: 75–90%, 4: > 90%) by two specialists in nuclear medicine and assessed.

**Results:**

The lung volume calculated from lung CT was 3749 ± 788 ml. The lung volume calculated from lung perfusion SPECT without using the breathing synchronization software was 3091 ± 610 ml. There was a significant difference between the lung volume calculated from CT and SPECT without using the breathing synchronization software (*P* < 0.01). The lung volume calculated from lung perfusion SPECT using the breathing synchronization software was 3435 ± 686 ml, and there was no significant difference between the lung volume calculated from CT and SPECT using the breathing synchronization software. The visual score improved with the use of breathing synchronization software (without software; 1.9 ± 0.6 vs. with software; 3.4 ± 0.7, *P* < 0.001).

**Conclusion:**

This study demonstrated that the breathing synchronization software could improve conformity between lung perfusion SPECT and lung CT.

## Background

The annual incidence of acute pulmonary embolism (PE) ranges from 500, 000 to 600,000 cases. Additionally, death due to PE ranges from 100,000 to 150,000 cases (Jamieson and Kapelanski 2000; Moser et al. 1990). Though anticoagulant therapy can reduce the risk of death, unnecessary therapy can increase the risk of bleeding. Therefore, it is important to diagnose and evaluate the grade of PE at an early stage.

Contrast lung computed tomography (CT) plays an important role in the diagnosis and evaluation of PE (Bloomgarden and Rosen 20012; Mayo et al. 1997). However, contrast lung CT is contraindicated in patients with renal dysfunction, multiple myeloma, or allergies to intravenous (IV) contrast (Kumar et al. 2015). Radionuclide imaging of lung ventilation and perfusion (V/Q) offers an alternative approach to diagnosing PE in these patients (McNeil et al. 1974; Gottschalk et al. 2002). However, the limited angular sampling and overlap of structures in planar images make it difficult to compare regional ventilation with regional perfusion, and V/Q scanning is a lengthy procedure. In recent years, a software-based hybrid diagnostic modality combining lung perfusion single-photon emission computed tomography (SPECT) and CT has been developed to expand the armamentarium for the diagnosis of PE (Kumar et al. 2015; Lu et al. 2014). However, generally, the conformity between SPECT and CT at the bottom of the lung is generally low because SPECT and CT have different acquisition timings. Commonly, the acquisition timing of SPECT is free breathing and that of CT is deep intake. At that point, there was no supporting software. Therefore, we developed a breathing synchronization software, which converted the original SPECT data into intake SPECT data by calculating the enlargement ratio using intake and free lung planar images. This study investigated the progression of conformity between lung perfusion SPECT and lung CT using the breathing synchronization software.

## Materials and methods

### Patient population

**Fig. 1:**
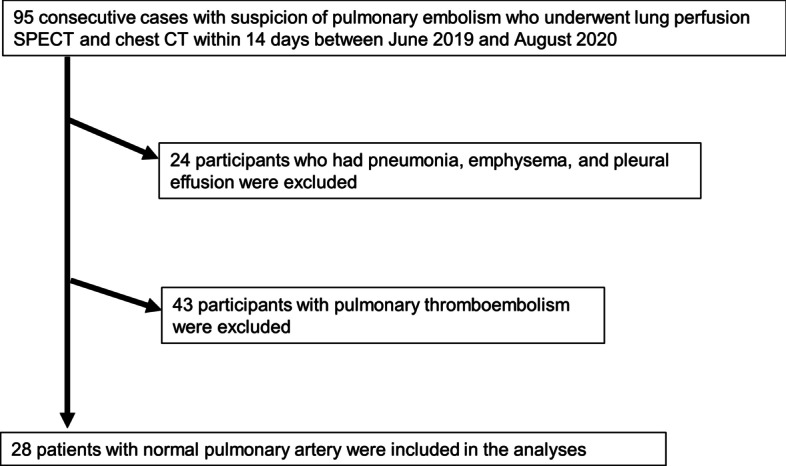
Flowchart of patient inclusion and exclusion criteria in the study. Abbreviations: CT = computed tomography; SPECT = single-photon emission computed tomography

Among 95 consecutive patients who underwent lung perfusion SPECT and lung CT within 14 days because of suspected PE between June 2019 and August 2020 in department of cardiovascular medicine, we identified 28 patients (73 ± 10 years) who had normal pulmonary artery on contrast lung CT (Fig. 1). The institutional review board approved this retrospective study, and the requirement to obtain informed consent was waived (M20076).

### Lung planar scan and perfusion SPECT

Lung planar and lung perfusion SPECT was performed using a dual-head gamma camera (Infinia, GE Healthcare, Buckinghamshire, UK) equipped with low-energy, high-resolution collimators. Planar images were recorded for 10 s each in the anterior and posterior views at deep-intake and free-breathing conditions after injecting 370 MBq of technetium-99m-macroaggregated albumin (^99m^Tc-MAA) with the patient in a supine position. Subsequently, SPECT data were acquired with 72 projections over an orbit of 360 degrees per step and 15 s per projection in free-breathing conditions. The image matrix size was 128 × 128. The image reconstruction was performed using filtered back projection and processed with Butterworth prefiltering (critical frequency 0.5, power 10.0).

### Chest CT

Data from chest CT performed within 14 days before and after lung perfusion SPECT were used (Aquilion Precision; Canon medical systems, Tokyo, Japan). Patients were scanned in the supine position with the following acquisition parameters: tube voltage, 100 kV at auto mA; rotation time, 0.5 s; collimation, 0.25 × 160 mm; and pitch, 1.381. Patients received IV injections of 80 ml Omnipaque-350 contrast at 3.5–4.5 ml/s via IV access, followed by a 40 ml saline flush. Individual contrast optimization was achieved by using a 20 ml test bolus in the right ventricle with a trigger level of 150 HU. An additional delay of 11 s was added before image acquisition in every examination. All scans were reconstructed as 2.0-mm-thick slices with an increment of 2.0 mm.

### Breathing synchronization software

#### Step 1

**Fig. 2:**
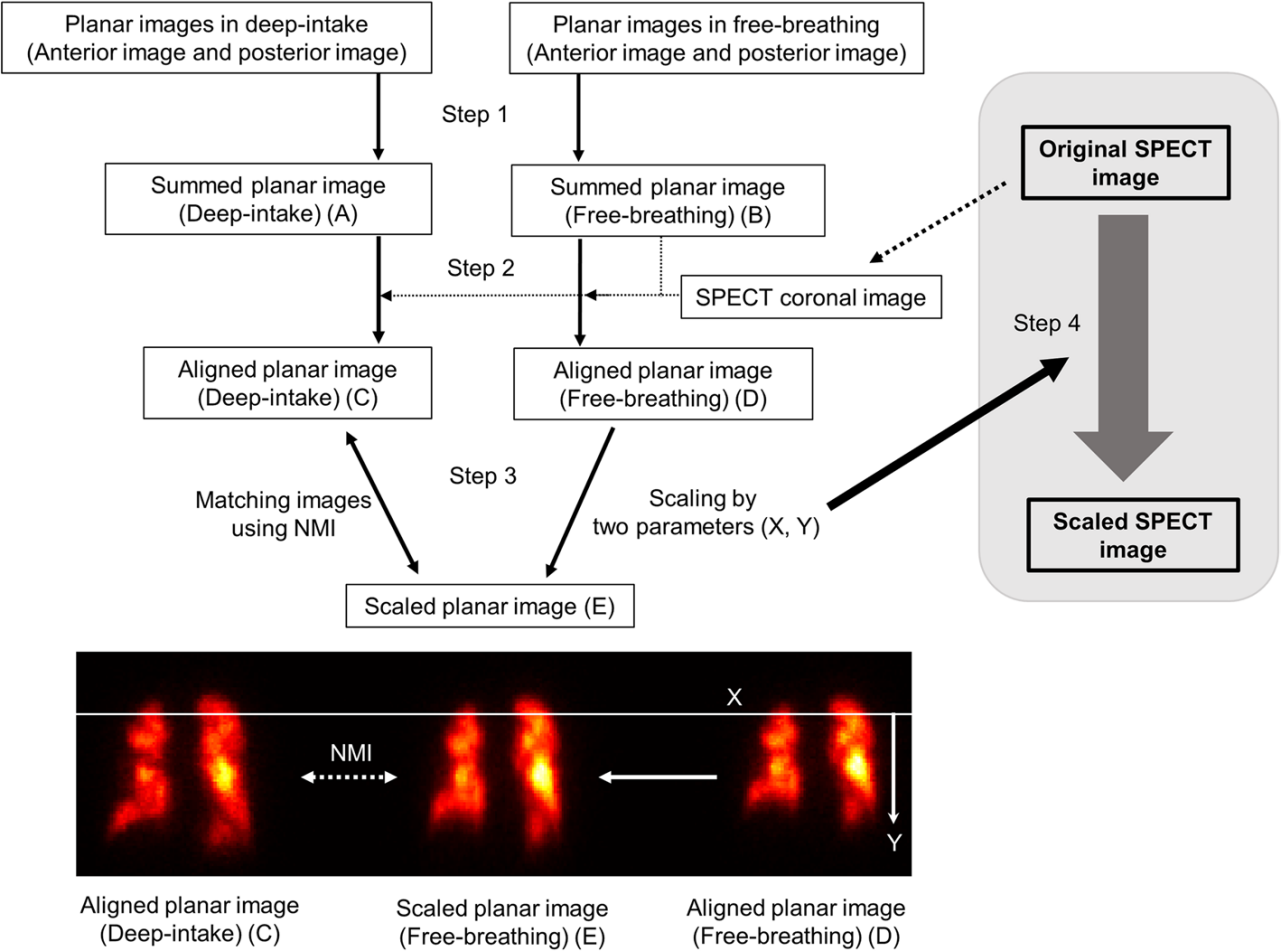
Algorism of the breathing synchronization software. The scaled and co-registered SPECT image was generated from the original SPECT image and planar images using the breathing synchronization software. Abbreviations: CT = computed tomography; NMI = normalized mutual information; and SPECT = single-photon emission computed tomography

In this step, the original anterior and posterior planar images were summed into one planar image to perform in the step that followed. The summed planar image at deep intake (A) was created from the original anterior and posterior planar images. The summed planar image at free breathing (B) was created from the original anterior and posterior planar images (Fig. 2 Step 1).

#### Step 2

In this step, planar images matched the pixel and matrix size with that of the original SPECT coronal image by aligning the images. The aligned planar image at deep intake (C) was created by aligning image A with the original SPECT coronal image as a reference, using rigid-body transformation with the cost function of mean squared error. The aligned planar image at free breathing (D) was created from image B with the same parameters as rigid-body transformation (Fig. 2 Step 2).

#### Step 3

In this step, the two optimized variables (*X* and *Y*) were calculated for scaling from the aligned planar image at free breathing to the aligned planar image at deep intake. The scaled planar image (E) was created from image D and deformed using the following two scaling parameters: *X*, which is the baseline position to scale image D, and *Y*, which is the magnification value to scale image D below *X*. The two parameters (*X* and *Y*) were calculated to maximize the agreement between image C as a reference and image E using normalized mutual information (NMI) (Fig. 2 Step 3).

#### Step 4

The scaled SPECT image was created from the original SPECT image and deformed using the two parameters (*X*, *Y*) (Fig. 2 Step 4).

### Assessment of conformity between lung perfusion SPECT and lung CT and statistical analysis

**Fig. 3:**
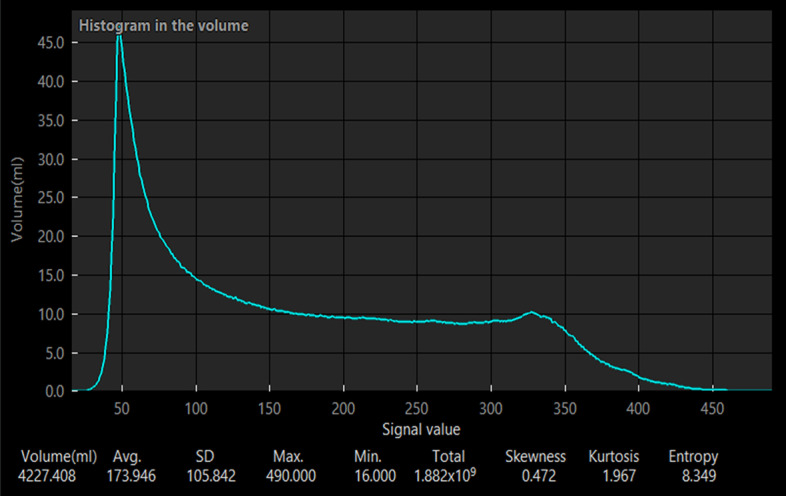
A histogram of radioactivity volume generates from ^99m^Tc-MAA SPECT. The x-axis represents the count from ^99m^Tc-MAA SPECT. The y-axis represents the volume per count. The lung volume was measured as the percentage area with radioactivity higher than the cutoff level of 10% of the maximum count on a histogram. In this case, the count at the edge of the lung perfusion SPECT was 49. Therefore, the total lung volume was calculated by adding up the volume per count from 49 to max count. Abbreviations: ^99m^Tc-MAA SPECT = technetium-99m-macroaggregated albumin single-photon emission computed tomography

Lung perfusion SPECT images and chest CT images were analyzed using a workstation (SYNAPSE VINCENT; FUJIFILM Medical Co., Ltd, Tokyo, Japan). The lungs volume was calculated from the original and scaled lung perfusion SPECT. The scaled lung perfusion SPECT was obtained using the breathing synchronization software. The edge of the lung perfusion SPECT was determined at 5–20% of the maximum counts using a histogram (Fig. 3). Lung volume as the gold standard was defined as the lung volume seen on chest CT. All lung volumes were calculated using the workstation. We compared the lung volumes, including those calculated from the original lung perfusion SPECT and the lung perfusion SPECT using the breathing synchronization software (the scaled SPECT image in each cutoff of the maximum counts) and lung CT using Dunnett’s test. Visual conformity between the lung SPECT images obtained with and without the use of the software and the lung CT image was scored 0–4 (0: 0–25%, 1: 25–50%, 2: 50–75%, 3: 75–90%, 4: > 90%) by two specialists in nuclear medicine and assessed. The Mann–Whitney U test was used to compare the scores between the original lung perfusion SPECT and the lung perfusion SPECT performed using the breathing synchronization software, the scaled SPECT image.

## Results

Patient characteristics are presented in Table 1. The mean age of the 28 patients was 73 ± 10 y, and 10 of them (36%) were men. The lung volume calculated using lung CT was 3749 ± 788 ml (Table 2) and that calculated from lung perfusion SPECT without using the breathing synchronization software was 3091 ± 610 ml, and there was a significant difference between lung volume calculated from CT and the original lung perfusion SPECT (*P* < 0.01) (Table 3). The lung volume calculated from lung perfusion SPECT using the breathing synchronization software with 10% of maximum counts was 3435 ± 686 ml, similar to the volume calculated from CT (Table 3). The visual score calculated using the breathing synchronization software improved (*P* < 0.001) (Table 4).

**Table 1:** Patient characteristics

	Total *N* = 28 (%)
Age (years)	73 ± 10
Male	10 (36)
Obesity (BMI ≥ 25 kg/m^2^)	9 (32)
Diabetes mellitus	9 (32)
Hypertension	17 (61)
Dyslipidemia	12 (43)
Smoking	11 (39)
CKD (eGFR < 60 mL/min/1.73m^2^)	10 (36)

BMI, body mass index; CKD, chronic kidney disease

**Table 2:** The volume of lung calculated using CT and lung perfusion SPECT

	Total (*n* = 28, ml)
CT	3749.1 ± 788.2
Original SPECT	3091.5 ± 610.4
SPECT using software (cutoff 10%)	3434.6 ± 685.8
SPECT using software (cutoff 5%)	5234.6 ± 1066.7
SPECT using software (cutoff 15%)	2835.1 ± 575.7
SPECT using software (cutoff 20%)	2439.6 ± 502.7

CT, computed tomography; SPECT, single-photon emission computed tomography

**Table 3:** Conformity of the lung volume between calculated from CT and SPECT using Dunnett’s test

	*P*
CT-Original SPECT	0.004
CT-SPECT using software (cutoff 5%)	< 0.001
CT-SPECT using software (cutoff 10%)	0.353
CT-SPECT using software (cutoff 15%)	< 0.001
CT-SPECT using software (cutoff 20%)	< 0.001

CT, computed tomography; SPECT, single-photon emission computed tomography

**Table 4:** Visual assessment

	Before using software	After using software	*P*
Interpreter 1	1.9 ± 0.6	3.3 ± 0.7	< 0.001
Interpreter 2	1.9 ± 0.5	3.5 ± 0.6	< 0.001

### Case presentation

**Fig. 4:**
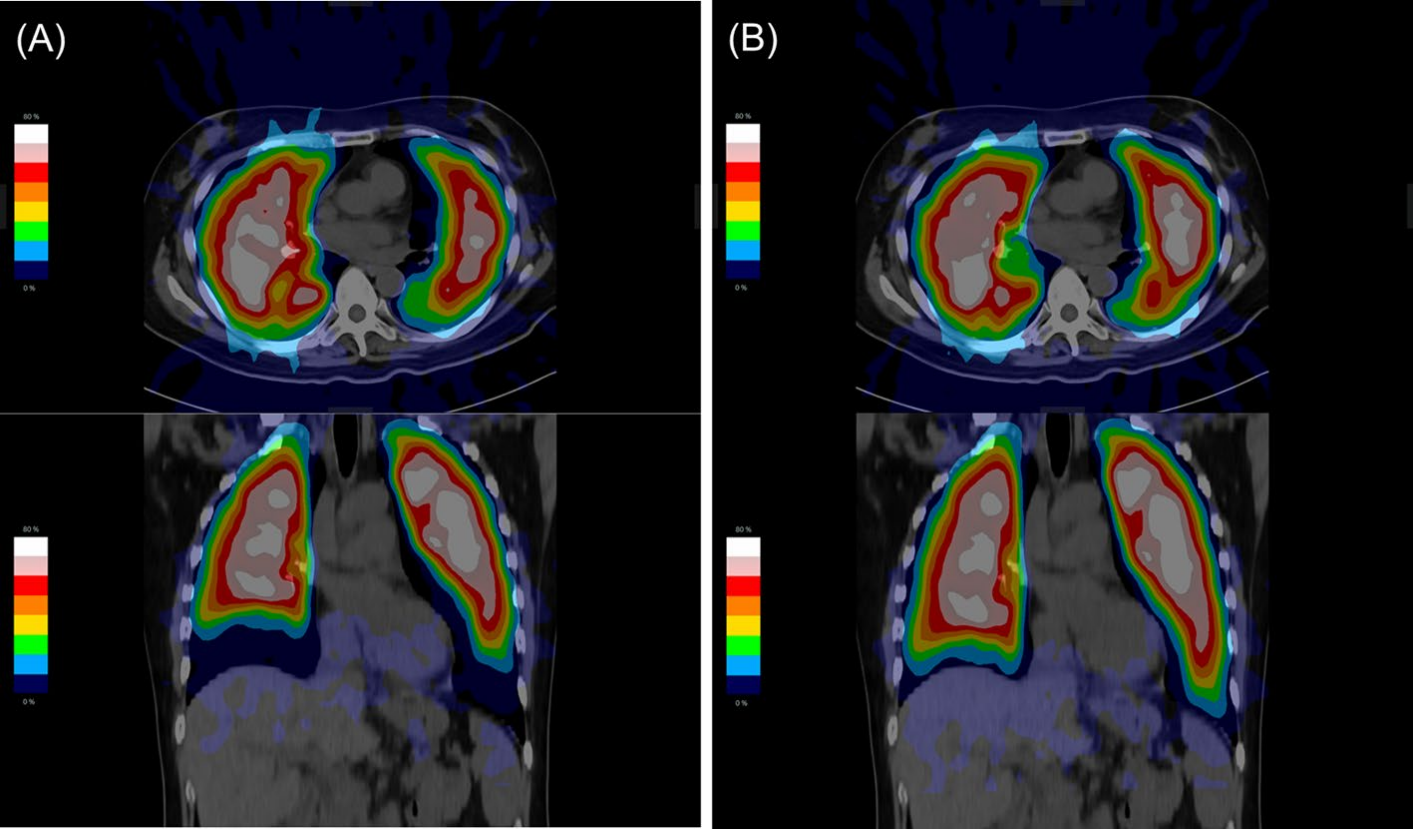
The fusion images between SPECT and CT before and after using the breathing synchronization software. A 50-year-old woman underwent lung perfusion SPECT and CT due to suspected PE; she had no abnormal morphological findings on the CT image and the lung perfusion SPECT image revealed normal lung perfusion. The lung volume calculated using CT was 4332 ml. **A** Before using the breathing synchronization software, the lung volume calculated using the original lung perfusion SPECT was 3351 ml; **B** after using the breathing synchronization software, the lung volume calculated using lung perfusion SPECT with the breathing synchronization software was 3974 ml; the volume of lungs calculated from SPECT with the breathing synchronization software was increased, and the conformity between lung perfusion SPECT and lung CT was improved. Abbreviations: CT = computed tomography; PE = pulmonary embolism; and SPECT = single-photon emission computed tomography

Figure 4 shows the original lung perfusion SPECT and CT fusion image and lung perfusion SPECT using breathing synchronization software and CT fusion image of a patient who underwent lung perfusion SPECT and CT due to suspected PE. The patient had no abnormal morphological findings on the CT image and had normal lung perfusion on the lung perfusion SPECT image. This 50-year-old woman had a history of hypertension and chronic kidney disease. The lung volume calculated using CT, from the original lung perfusion, and from lung perfusion SPECT using the breathing synchronization software was 4332 ml, 3351 ml, and 3974 ml, respectively. The visual score of the original lung perfusion SPECT and lung perfusion SPECT using the breathing synchronization software was 1.5 and 4.0, respectively. The lung perfusion SPECT image obtained using the breathing synchronization software was more similar to the CT image than the original lung perfusion SPECT image.

## Discussion

In the present study, our findings demonstrated that the conformity between SPECT and CT images progressed using the breathing synchronization software.

Given the high-risk nature of PE, evaluation of its severity using imaging modalities is essential. Computed tomographic pulmonary angiography (CTPA) and V/Q scans are commonly used to diagnose PE. However, besides impaired renal function or contraindication to iodine-containing IV contrast, approximately 6–8% of CTPA studies are considered nondiagnostic (Stein et al. 2006; Jones and Wittram 2005; Abujudeh et al. 2009). Recently, techniques have rendered CTPA too sensitive, resulting in the overdiagnosis and overtreatment of PE. This has led to the discovery of clinically unimportant PE and exposed patients to potential harm from unnecessary treatment (Sheh et al. 2012; Schissler et al. 2013). However, V/Q scans are commonly used in patients with contraindications to CTPA, and the relatively high rate of indeterminate V/Q scans, especially in patients with airway disease, limits the clinical utility (Sostman et al. 2008). In recent years, Q-SPECT/CT has been shown to improve diagnostic rates. Kumar N et al. reported that while over 40% of planar V/Q examinations had indeterminate interpretations, only 4.9% of Q-SPECT/CT scans were considered indeterminate (Kumar et al. 2015). Several studies have reported that the pulmonary vascular obstruction measured on lung perfusion scintigraphy could be an independent risk factor for PE recurrence and chronic thromboembolic pulmonary hypertension (CTEPH) (Tromeur et al. 20182018; Pesavento et al. 2017; Planquette et al. 2006; Wartski and Collignon 2006; Sanchez et al. 2010; Meneveau et al. 1988; Miniati et al. 2006). Pesavento et al. reported that recurrent venous thromboembolism and/or chronic thromboembolic pulmonary hypertension developed in 34 out of the 324 patients (10.5%) with residual pulmonary obstruction evaluated using perfusion lung scanning (hazard ratio: 2.26, 95% CI 1.23–4.16). Therefore, it is important to diagnose PE and to evaluate its severity using scintigraphy. For these reasons, Q-SPECT/CT is a simple method for diagnosing PE and is useful in clinical practice. However, in many cases, the conformity between SPECT and CT images is low, especially in the lower lobe of the lung. This is because of the influence of breathing and the difference in the timing and duration of data acquisition between SPECT and CT. In the current study, the use of breathing synchronization software improved the conformity between SPECT images and CT images. Therefore, Q-SPECT/CT using the breathing synchronization software could have not only diagnostic value for PE but also prognostic value for PE recurrence and exacerbation of CTEPH.

### Study limitations

This study has some limitations. First, the number of patients was relatively small, which limited the statistical reliability of the study. However, our results demonstrated that the lung volume calculated using SPECT was significantly correlated with that calculated using CT. Second, the cutoff level of the maximum radioactivity value in ^99m^Tc-MAA SPECT image analysis for calculating the lung volume did not reach a consensus. This study investigated cutoff levels from 5 to 20%, and 10% was the most suitable cutoff level. However, further studies are necessary to confirm the adequacy of this cutoff level. Third, in this study, we did not investigate the breathing synchronization software’s effect on improvement of the diagnosis of PE. Therefore, further prospective studies with a large population are needed to confirm the diagnostic value of the breathing synchronization software.

## Conclusion

This study demonstrated that breathing synchronization software could improve conformity between lung perfusion SPECT and lung CT in patients with suspected PE.

## Data Availability

The datasets used and analyzed during the current study are available from the corresponding author on reasonable request.

## Abbreviations

BMI: Body mass index
CKD: Chronic kidney disease
CT: Computed tomography
CTEPH: Chronic thromboembolic pulmonary hypertension
CTPA: Computed tomographic pulmonary angiography
IV: Intravenous
NMI: Normalized mutual information
PE: Pulmonary embolism
SPECT: Single-photon emission computed tomography
^99m^Tc-MAA: Technetium-99m-macroaggregated albumin
V/Q: Lung ventilation and perfusion
